# DMCM: Dwo-branch multilevel feature fusion with cross-attention mechanism for infrared and visible image fusion

**DOI:** 10.1371/journal.pone.0318931

**Published:** 2025-03-28

**Authors:** Xicheng Sun, Fu Lv, Yongan Feng, Xu Zhang

**Affiliations:** 1 Software College, Liaoning Technical University, Huludao, Liaoning, China; 2 Department of Basic Teaching School of Software, Liaoning Technical University, Huludao, Liaoning, China; Anhui University, CANADA

## Abstract

In response to the limitations of current infrared and visible light image fusion algorithms—namely insufficient feature extraction, loss of detailed texture information, underutilization of differential and shared information, and the high number of model parameters—this paper proposes a novel multi-scale infrared and visible image fusion method with two-branch feature interaction. The proposed method introduces a lightweight multi-scale group convolution, based on GS convolution, which enhances multi-scale information interaction while reducing network parameters by incorporating group convolution and stacking multiple small convolutional kernels. Furthermore, the multi-level attention module is improved by integrating edge-enhanced branches and depthwise separable convolutions to preserve detailed texture information. Additionally, a lightweight cross-attention fusion module is introduced, optimizing the use of differential and shared features while minimizing computational complexity. Lastly, the efficiency of local attention is enhanced by adding a multi-dimensional fusion branch, which bolsters the interaction of information across multiple dimensions and facilitates comprehensive spatial information extraction from multimodal images. The proposed algorithm, along with seven others, was tested extensively on public datasets such as TNO and Roadscene. The experimental results demonstrate that the proposed method outperforms other algorithms in both subjective and objective evaluation results. Additionally, it demonstrates good performance in terms of operational efficiency. Moreover, target detection performance experiments conducted on the $M^{3}FD$ dataset confirm the superior performance of the proposed algorithm.

## Introduction

Multi-sensor images offer a way to capture complementary information of the same scene, facilitating enhanced visual understanding and scene perception, and overcoming the limitations of single-sensor imaging. By synthesizing information from various sensors, such as infrared and visible light, these images can provide more robust data for subsequent image processing or decision-making tasks [1]. Specifically, the fusion of infrared and visible light images has become a critical area of study within computer vision. Infrared-visible light fusion is now widely applied across many fields [2]. Visible light sensors, which rely on reflected light, offer high spatial resolution and detailed background information; however, they often fail to capture targets effectively in poor lighting conditions or when objects are camouflaged. In contrast, infrared sensors detect the thermal radiation emitted by objects, are unaffected by lighting or environmental challenges, and can operate continuously, day or night. Therefore, fusing infrared and visible light images into a single, cohesive image is vital for retaining critical information from both modalities.

In recent decades, various traditional infrared and visible image fusion methods have been proposed and have demonstrated strong performance. These methods generally fall into four major categories: multiscale transform-based methods, sparse representation-based methods, subspace-based methods, and hybrid models. The multiscale methods include wavelet transform techniques [3] and non-subsampled contourlet transform [4]. Sparse representation methods [5] and [6] focus on constructing an overcomplete dictionary from high-quality natural images, enabling sparse representation of both infrared and visible light images, thereby enhancing the final fused image. Subspace-based methods, such as principal component analysis (PCA) [7] and independent component analysis (ICA) [8], project high-dimensional source images into lower-dimensional subspaces, capturing their intrinsic structures more effectively. Hybrid models, which combine the strengths of multiple techniques, have also been proposed. For example, Liu [9] introduced a unified fusion framework by integrating multiple fusion approaches to improve overall performance.

In the task of infrared and visible light image fusion, [10] applyed complex evidence theory for multi-source data fusion is an effective approach. [11] combined attention mechanisms with long and short time sequences allows for adaptive learning of the model’s state estimation. [12] proposes an infrared and visible light video fusion algorithm based on possibility distribution synthesis theory. By quantitatively describing feature differences, correlation measures, and joint synthesis, it achieves better target and detail preservation, significantly improving fusion quality. [13] innovates 3D object detection by fusing multiple sensors and discusses the limitations of current methods, as well as future research directions.

In recent years, convolutional neural networks (CNNs)-based fusion methods have gained prominence due to their superior ability to represent features. Generative Adversarial Networks (GANs) have also been employed for image fusion, due to their powerful unsupervised distribution estimation capabilities. Most Transformer-based studies have concentrated on capturing the global information of images, aiming to address the limitations of convolutional operations, which primarily capture local features. However, the traditional self-attention mechanism is not suitable for outdoor applications due to its high computational complexity, resulting from quadratic multiplication and a large number of model parameters. Moreover, the Transformer architecture often fails to fully utilize differential and shared information between multimodalities, leading to suboptimal feature extraction. Similarly, standalone CNNs struggle to capture long-range dependencies, and their ability to extract feature scale and texture information is limited.

Traditional algorithms rely on manually designed feature templates, which fail to adapt to complex and diverse image scenarios. Especially in noisy or variable lighting conditions, the extracted features often lack robustness. While deep learning models have powerful capabilities for automatic feature learning, they exhibit limitations in extracting high-frequency features (e.g., edges or textures). In particular, deeper network layers tend to overlook local details, leading to the smoothing of high-frequency features. Traditional methods for texture retention often employ direct fusion or simple weighting, resulting in blurred image edges, distorted texture structures, and “ghosting” effects in edge regions. Deep learning algorithms may suffer from overfitting or limitations in feature extraction layers, resulting in the loss of local textures or blurring of details when generating high-resolution outputs. Especially in low-contrast or complex textured areas, the generated images may fail to accurately reflect the target’s texture characteristics. High-quality feature extraction is foundational for subsequent tasks such as object detection, classification, registration, or fusion. Insufficient or indistinct feature extraction compromises the overall performance of algorithms. In infrared and visible image fusion, insufficient texture information can obscure target areas, affecting scene interpretability or the recognition of critical targets. In practical applications such as nighttime license plate recognition and road crack detection, algorithms must not only extract functional features but also preserve overall image details to ensure usability.

Infrared and visible images capture different key information (e.g., infrared highlights thermal radiation, while visible light retains rich texture details). Traditional fusion methods often involve simple feature weighting or merging, which may overlook the complex relationships between the features of the two modalities. The cross-attention mechanism computes interdependencies among features, effectively capturing the complementary information across modalities and thereby enhancing the quality of the fusion results. Cross-attention not only focuses on local details but also dynamically adjusts to global features, which is crucial in complex scenarios (e.g., intricate backgrounds or sparse target information). This capability helps prevent issues like information loss or over-smoothing.Traditional methods often rely on fixed weight allocations, which are insufficient for adapting to diverse image content. Many approaches focus solely on low-level features, neglecting the integration of high-level semantic information, which results in subpar visualization of the fused image. Some methods merely combine features without thoroughly exploring the dynamic correlations between modalities. Cross-attention learns the dynamic dependencies between the features of both modalities, enhancing the fusion effect by emphasizing relevant regions.Our proposed cross-attention mechanism effectively integrates modal features, offering a novel approach to infrared and visible image fusion. It enhances the interpretability and applicability of fusion algorithms, addressing the deficiencies of existing methods in adaptability and dynamism.

To address these issues, this paper presents a multi-scale image fusion algorithm with two-branch, multi-level feature fusion. To acquire global context and multi-scale feature information while reducing the number of network parameters, a lightweight multi-scale grouped convolution(LMGC) is introduced. To effectively extract and fuse the differential and shared information from multimodal images, a lightweight cross-attention fusion module (LCFM) is proposed. Additionally, to capture spatial information and enhance multi-level feature fusion and texture extraction, an improved multi-dimensional hybrid spatial attention (MHSA) mechanism is developed, along with an optimized dual-branch multi-level fusion module (DMFM). Our contributions can be summarized in four aspects:

1. We propose a two-branch multilevel feature fusion with the cross-attention mechanism for infrared and visible image fusion(DMCM)
2. We propose a lightweight multi-scale grouped convolution based on GS convolution and introduce Swiftformer to extract and fuse global and local features images fully.
3. The improved dual-branch multi-level fusion module is designed to capture rich multi-level features and fully extract texture information, with one branch employing lightweight cross-attention fusion and multi-dimensional hybrid spatial attention, and the other leveraging a Sobel operator and deep convolution for edge information extraction.
4. Extensive experiments on public datasets demonstrate that the proposed model outperforms seven classical algorithms in terms of subjective and objective evaluations, and operational efficiency. Furthermore, the proposed model achieves excellent performance in downstream tasks such as target detection.

The rest of the paper is organized as follows: Sect 2 discusses existing deep learning-based methods for the fusion of infrared and visible images. In Sect 3, we present the overall network architecture of the proposed algorithm, a detailed explanation of the proposed modules, the loss function. Sect 4 describes the experimental parameters, training details, experiments comparing the algorithm of this paper with other state-of-the-art algorithms, ablation experiments and target detection experiments. Finally, we give conclusions in Sect 5.

## Related works

Liu [14] first introduced CNNs for image fusion using twin neural networks to generate weight mappings for fusion. Li [15] proposed DenseFuse, an encoding-decoding-based method with dense connections, to fuse infrared and visible images, aiming to mitigate the loss of deep features in the fusion process. Jian [16] incorporated an attention mechanism into a symmetric otoacoustic emission network to enhance salient infrared information during fusion. Li [17] designed a learnable fusion network trained in two phases, which better preserved image details and addressed the limitations of traditional manual fusion strategies. Similarly, Ma [18] developed an end-to-end model that uses a salient target mask to guide network training, ensuring that thermal information is effectively highlighted. Li and Wu [19] were the first to employ an autoencoder network for infrared-visible image fusion (IVIF), incorporating dense blocks in the encoder to comprehensively extract features, followed by the application of additive in the fusion layer to generate fusion outputs.

Ma [20] initially used GANs to establish an adversarial process between visible images and fusion results, enhancing texture detail. However, this method relied solely on information from visible images, resulting in the loss of target contrast and contour. To address this issue, they later introduced a dual discriminator GAN [21], which utilized both high- and low-resolution versions of the fused image to deceive two discriminators. This approach integrated both infrared and visible image data, significantly improving fusion performance. Li [22] introduced an end-to-end GAN model that incorporates multi-class classification constraints to further enhance fusion. Liu [23] designed a fusion network featuring a single generator and dual discriminators, employing a saliency mask to preserve the structural information of infrared targets and the texture details from visible light.

The Transformer model was first introduced by Vaswani [24] to address challenges in natural language processing, where it achieved remarkable success. Building on this, Dosovitskiy [25] extended the Transformer architecture to the visual domain by designing the Vision Transformer (ViT) for image classification tasks. Owing to its self-attention mechanism, which effectively captures long-term dependencies, the Transformer has since been applied to a wide range of computer vision tasks, including object detection [26], video restoration [27], and image super-resolution [28]. These successes have catalyzed the development of Transformer-based methods within the field of image fusion. VS [29] were pioneers in proposing a Transformer model for image fusion, enabling the extraction of both local and long-range information through the use of spatial and Transformer branches. Ma [30] advanced this work by introducing a generalized fusion method that integrates cross-domain learning with the Swin Transformer. Their model effectively preserves foreground targets from thermal images and background textures from visible light images, given that these regions tend to exhibit higher pixel intensities in their respective modalities. Tang [31] proposed an end-to-end Transformer architecture for infrared and visible image fusion. Their design features a dual attention residual module to extract critical features from the source images, while a Transformer module is utilized to retain global complementary information, ensuring the preservation of long-range dependencies.

## The proposed method

### Framework overview

**Fig 1:**
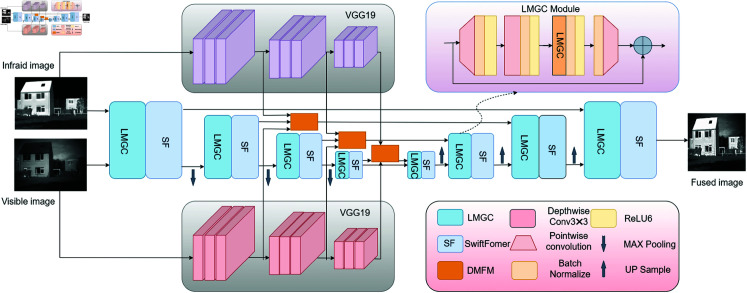
The framework of method we proposed (DMCM).

Our network is composed of three primary components: feature encoding, feature fusion, and feature decoding, as illustrated in Fig 1. To effectively extract deep features from both infrared and visible images, we employ UNet [32] as the backbone network, using concatenated infrared and visible images as input. The backbone’s feature extraction stage incorporates lightweight multi-scale grouped convolution and SwiftFormer modules to capture local and global features, respectively. For shallow feature extraction, we utilize a pre-trained VGG19 [33] as a secondary stem within the feature extraction architecture. In the feature fusion stage, a dual-branch multilevel fusion module is introduced to integrate deep and shallow features more comprehensively.

### Feature extraction portion of the backbone network

#### LMGC.

**Fig 2:**
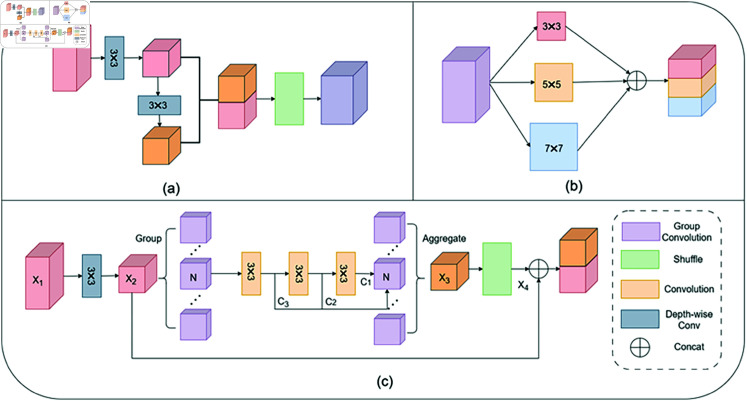
Lightweight multi-scale grouped convolution (LMGC) and GSconv legend: (a) GSConv (b) multi-scale feature extraction (c) Lightweight multi-scale grouped convolution.

To minimize feature information loss and reduce the number of network parameters, Li [34] introduced GSConv (Fig 2(a)), which combines depthwise separable convolution with a channel mixing operation. Depthwise separable convolution not only captures local feature information but also significantly reduces the number of network parameters. The channel mixing operation facilitates interaction and communication between different channels, thereby enhancing the richness and diversity of feature representations.

Building on GSConv, this paper proposes the LMGC (Fig 2(c)), which extracts feature information across different scales while further reducing network parameters. Grouped convolution, an advanced convolutional algorithm, divides input channels into groups and performs independent convolution operations within each group. This strategy reduces the model’s parameter count by limiting the number of channels processed by each convolution kernel, thereby decreasing storage requirements, mitigating the risk of overfitting, and improving the model’s generalization capabilities. In this study, grouped convolution replaces depthwise separable convolution to achieve model lightweighting.

This module addresses the limitations of current algorithms in capturing multi-scale information. By incorporating multi-scale receptive field designs into grouped convolutions, we significantly enhance the model’s adaptability to various resolutions and complex structures while reducing parameter counts and computational overhead. This approach enables substantial reductions in hardware requirements while maintaining model accuracy. Compared to existing convolutional methods, it more comprehensively extracts both local image details (e.g., textures) and global semantic information (e.g., large-scale structures) without compromising computational efficiency.

Inspired by VGG , which expands the receptive field by stacking small convolution kernels—using two 3x3 convolutions to achieve a 5x5 receptive field and three 3x3 convolutions to approximate a 7x7 receptive field—this paper cascades three 3x3 convolution kernels with the addition of multiple short connections to enable multi-scale feature extraction (Fig 2(b)).

Initially, the infrared and visible images are concatenated, and channel dimensionality reduction is performed using 1x1 pointwise convolution to halve the number of channels. This process can be expressed as:


$$\begin{array}{c} X=PWConv\left(Concate\left(IR,VIS\right)\right) \end{array}$$ (1)

where IR and VIS represent the source images, Concate denotes the concatenation operation, and PWConv refers to pointwise convolution. Next, the input features are partitioned into several groups, and multi-scale information is extracted using cascaded 3x3 convolution kernels:


$$\begin{array}{c} X_{2}=Group\left(X_{1}\right) \end{array}$$ (2)

$$\begin{array}{c} X_{3}^{N}=Concate\left(C_{1}.C_{2}.C_{3}\right) \end{array}$$ (3)

where Group refers to grouped convolution, $X_{3}^{N}$ indicates one of the N divided parts, and represent the output after one, two, and three 3x3 convolutions, respectively. Subsequently, the sub-modules of each group are recombined, and channel independence is broken using the channel mixing operation, which promotes information sharing between groups and enhances inter-channel interaction:


$$\begin{array}{c} X_{4}=Shuffle\left(Aggregate\left(X_{3}\right)\right) \end{array}$$ (4)

where Aggregate refers to the recombination of grouped convolutions, and Shuﬄe denotes the channel shuﬄe operation. Finally, the original features are concatenated with the input to retain more detailed information.


$$\begin{array}{c} LMGC\left(IR,VIS\right)=Concate\left(X_{1},X_{4}\right) \end{array}$$ (5)

#### Swiftformer.

Given the significant computational overhead of traditional attention mechanisms, SwiftFormer, proposed in [35], offers an efficient alternative. By eliminating key-value interactions while retaining performance, SwiftFormer encodes query-key interactions through merged linear projection layers. This approach, termed effective additional attention, results in faster inference and more robust contextual representations. To address the limited ability of our base model in capturing global context, we incorporate the SwiftFormer module as the global feature extraction component in the backbone network. The fusion process can be formulated as:


$$\begin{array}{c} F_{L}=LMS-GC\left(IR,VIS\right) \end{array}$$ (6)

$$\begin{array}{c} F_{LS}=Swiftformer\left(F_{L}\right) \end{array}$$ (7)

where IR and VIS represent the two input source images, $F_{L}$ denotes the local feature maps output by the LMS-GC, and $F_{LS}$ represents the local-global feature maps output by the SwiftFormer module.

### Feature fusion module

#### DMFM.

**Fig 3:**
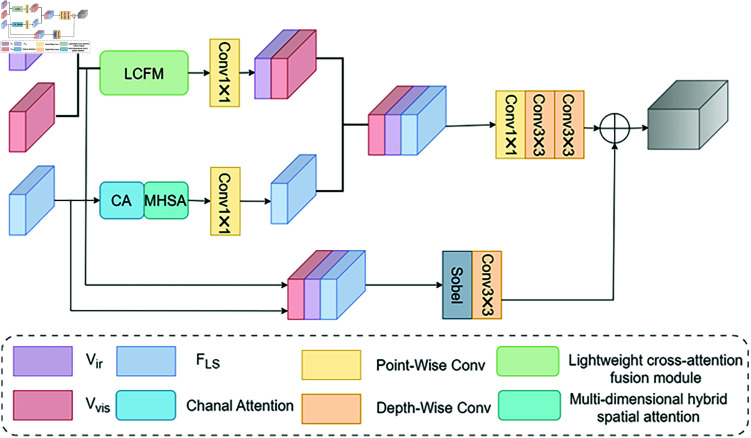
Dual-branch multilevel fusion module (DMFM).

To effectively fuse shallow and deep features from the two modalities, we design a dual-branch multilevel fusion module based on multilevel attention [36] (see Fig 3). To compensate for the lack of spatial information in deep features, we introduce multidimensional hybrid spatial attention after the channel attention (CA) mechanism. Each deep feature map is represented as layers $F_{LS}^{1}$, $F_{LS}^{2}$,$F_{LS}^{3}$ ,$F_{LS}^{4}$ with channels of 32, 64, 128, and 256. Ascending pointwise convolution increases the number of channels in each layer to 64, 128, 256, and 512, while descending pointwise convolution reduces the channel dimensions back to 32, 64, 128, and 256. For this task, we select two VGG-19 networks with pre-trained weights as sub-stem networks. These networks take visible and infrared images as input and aim to fully exploit the shallow features of the source images.

The module is specifically designed to resolve mismatches in cross-domain information fusion encountered by current algorithms. It captures salient features from different information domains and achieves more effective fusion. This design addresses the issue of information loss caused by single-domain fusion in traditional methods. Unlike conventional fusion methods that rely on direct weighting or simple concatenation, the module integrates features from different layers in a manner tailored to their characteristics.

To enhance the fusion of shallow infrared and visible light features, we employ a lightweight cross-attention fusion module. The infrared features from the sub-stem network are denoted as $V_{ir}^{1}$ ,$V_{ir}^{2}$,$V_{ir}^{3}$ ,corresponding to layers with 64, 128, and 256 channels, respectively. Similarly, the visible features are denoted as $V_{vis}^{1}$ ,$V_{vis}^{2}$,$V_{vis}^{3}$. Finally, the deep features, infrared features, and visible features are fused along the channel dimension.

The shallow feature maps from the infrared and visible images are first concatenated, and the fusion is performed using a lightweight cross-attention fusion module combined with 3x3 depthwise convolution. Concurrently, deep features undergo feature extraction using channel attention and multidimensional hybrid spatial attention. This process can be expressed as:


$$\begin{array}{c} f_{1}=PWConv\left(LCFM\left(V_{ir},V_{vis}\right)\right) \end{array}$$ (8)

$$\begin{array}{c} f_{2}=PWConv\left(MHSA\left(CA\left(F_{LS}\right)\right)\right) \end{array}$$ (9)

where denote the shallow features of the source images at the VGG-19 network output, $F_{LS}$ is the backbone network output, and DWConv represents the 3x3 depthwise convolution. To effectively fuse and enhance feature representation, the outputs from both sub-branches are concatenated, dimensionality is reduced using pointwise convolution, and detailed features are extracted via depthwise convolution. This process is expressed as:


$$\begin{array}{c} F_{1}=DPConv\left(Concate\left(f_{1},f_{2}\right)\right) \end{array}$$ (10)

where DPConv represents the pointwise convolution and two 3x3 depthwise convolutions. Finally, in the second branch, all feature maps are concatenated, and edge texture information is extracted using the Sobel operator and depthwise convolution. The two branches are then fused, with the final process represented as:


$$\begin{array}{c} F_{2}=DWConv\left(Sobel\left(Concate\left(V_{ir},V_{vis},F_{LS}\right)\right)\right) \end{array}$$ (11)

where Sobel represents the edge detection operator, denotes the final output feature map, and fusion represents the final integration of both branches.

#### MHSA.

To address the limitations of prior channel attention mechanisms, such as inadequate generalization ability and the issues arising from channel dimension reduction, efficient local attention(ELA) was proposed in [37]. ELA extracts feature vectors in both horizontal and vertical directions by utilizing banded pooling in the spatial dimension, while maintaining elongated kernel shapes to capture long-range dependencies. This approach also reduces the influence of irrelevant regions on label prediction.

**Fig 4:**
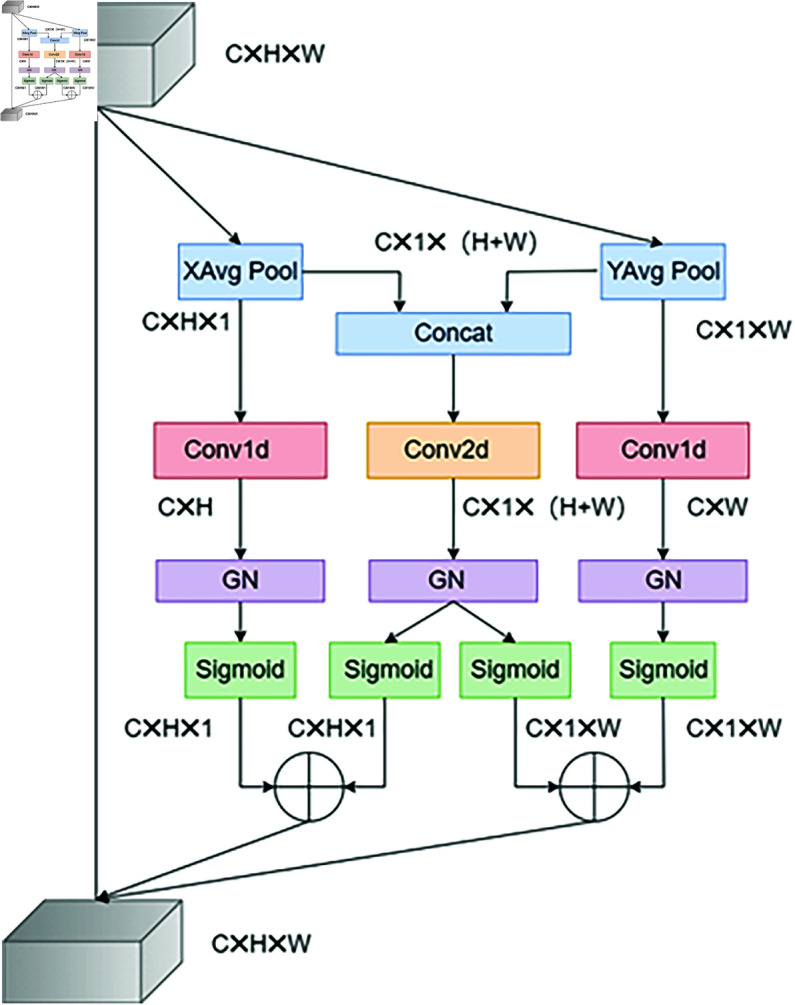
The spatial attention wo proposed (MHSA).

To mitigate the lack of spatial information, we propose a MHSA (Fig 4) based on ELA. While ELA convolves and normalizes the two dimensions separately before generating weights, which limits the diversity of information obtained, we introduce a multidimensional fusion branch to enhance feature representation. This addition improves model accuracy by fusing features across dimensions, thereby capturing richer and more comprehensive information.

Initially, the input features are pooled equally across both dimensions, processed using 1D convolution, and normalized. The corresponding weights are then generated via a Sigmoid function. The fusion branch then concatenates the pooled features, after which a 3x3 depthwise convolution extracts local features and reduces the number of network parameters. The output from the multidimensional fusion branch is divided into two parts, with corresponding weights generated through Sigmoid. Finally, these weights are fused with the weights of the other two dimensions and multiplied with the initial features to produce the final feature map.

#### LCFM.

The cross-attention mechanism [38], an extension of self-attention originally introduced in Transformers, enhances model performance by focusing not only on internal positional relationships within an input sequence but also on relationships between positions in different input sequences. While traditional self-attention [39] emphasizes internal relations within a single sequence, cross-attention plays a crucial role in multimodal fusion in computer vision, allowing the transfer of information between different modalities.

The cross-attention mechanism further enhances the fusion process by effectively combining high-temperature regions from infrared images with edge information from visible light images. It enables precise matching and interaction of cross-modal features, addressing the inefficiencies of current methods in fusing features from different modalities. Existing approaches often lack accurate feature interaction mechanisms during modality fusion, leading to feature conflicts or information redundancy. In contrast, cross-attention dynamically adjusts the focus on regions of interest in different modalities, significantly improving the efficiency and accuracy of cross-modal fusion.

**Fig 5:**
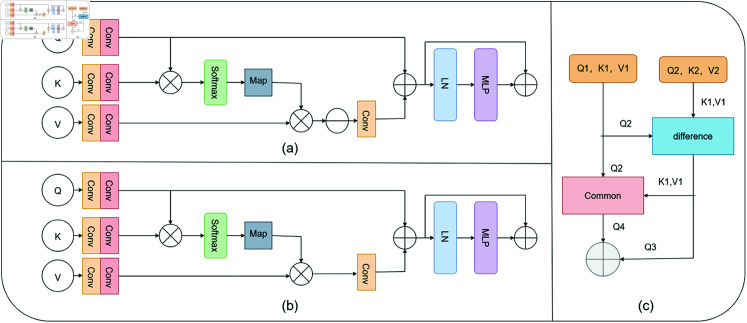
The light cross-attention fusion module (LCFM) legend: (a) Difference Feature Attention (b) Common Feature Attention (c) The LCFM we proposed.

In the context of infrared and visible image fusion, the objective is to produce a composite image that captures salient targets while preserving rich texture details. Fully utilizing the distinctive and shared information between source images is essential for superior fusion performance. Motivated by the effectiveness of cross-attention in extracting common features across images, we propose Difference Feature Attention in Fig 5(a) and Common Feature Attention in Fig 5(b), both of which are embedded into the LCFM, as depicted in Fig 5(c).

First, the marked segments are transformed into query Q, key K, and value V using depthwise convolution followed by pointwise convolution, as expressed by the following equations:


$$\begin{array}{c} Q=DPConv\left(Q\right),K=DPConv\left(K\right),V=DPConv\left(V\right) \end{array}$$ (12)

Next, to explore common information between infrared and visible images while accounting for long-term dependencies, we calculate the similarity matrix between Q and K using a dot-product attention layer. This matrix is then multiplied by V to infer the shared information between Q and V. This process is represented as:


$$\begin{array}{c} x=Soft\max\left(\frac{QK^{T}}{\sqrt{d}}\right)V \end{array}$$ (13)

Subsequently, the differential information can be obtained by subtracting the shared information from the original data, represented as:


$$\begin{array}{c} X_{2}=DWConv\left(V-X_{1}\right) \end{array}$$ (14)

Finally, to obtain complementary information from multimodal images, we inject the differential information back into Q, which can be formulated as:


$$\begin{array}{c} F=MLP\left(LN\left(X_{2}+Q\right)\right)+\left(X_{2}+Q\right) \end{array}$$ (15)

where LN represents layer normalization, and MLP refers to the multi-layer perceptron.

### loss fuction

The goal of image fusion is to provide an information-rich image with sufficient detail and balanced intensity by combining the favorable features of the source image. The loss function mainly consists of two parts, the fundamental loss and the contrast loss, the fundamental loss can be defined as.


$$\begin{array}{c} L=L_{pixel}+\alpha L_{grad} \end{array}$$ (16)

where $L_{pixel}$ and $L_{grad}$ denote the pixel loss and gradient loss, respectively. is the hyperparameter that balances these two loss terms.


$$\begin{array}{c} L_{grad}=\frac{1}{HW}{∥\nabla I_{f}-\max(\nabla I_{i}r\cdot \nabla I_{vis})∥}_{1} \end{array}$$ (17)

Where *∇* ⁡  denotes the Sobel operator, which is used to compute the gradient. $∥{∥}_{1}$ denotes the number of $L_{1}$ norms. H and W denote the height and width respectively. max( , ) denotes the element-by-element maximum selection.


$$\begin{array}{c} L_{pixel}=\frac{1}{HW}{∥I_{f}-\max(I_{ir},I_{vis})∥}_{1} \end{array}$$ (18)

Where $I_{ir}$, $I_{vis}$ are the source images. if is the fused image. The contrast loss can be expressed as:


$$\begin{array}{c} L_{i}r=\overset{N}{\underset{i=1}{\sum }}\frac{{∥r_{i}-r_{i}^{+}∥}_{1}}{\overset{M}{\underset{m}{\sum }}{∥r_{i}-r_{i}^{m-}∥}_{1}},L_{v}is=\overset{N}{\underset{i=1}{\sum }}\frac{{∥v_{i}-v_{i}^{+}∥}_{1}}{\overset{M}{\underset{m}{\sum }}{∥v_{i}-v_{i}^{m-}∥}_{1}} \end{array}$$ (19)

where N and M are the number of VGG layers and the number of negative samples for each positive sample, respectively. $r_{i}$ denotes the foreground feature of the fused image. m denotes the negative sample. $r_{i}^{+}$ and $r_{i}^{m-}$are the positive and negative samples. So the final loss function can be expressed as:


$$\begin{array}{c} L_{total}=L+L_{ir}+L_{vis} \end{array}$$ (20)

## Experiments

### Comparison with SOTA methods

#### Datasets and experimental parameters.

For our experiments, we utilize two publicly available datasets: the TNO dataset [40] and the Roadscene dataset [41]. The experiments are conducted in the following environment: an Intel i9-12900k processor (3.2 GHz), an NVIDIA GeForce RTX 3090 GPU, 64 GB of RAM, Python 3.9.0 as the programming language, Windows 11 as the operating system, PyTorch 1.10.1 as the deep learning framework, and CUDA version 11.2.

#### Training details.

The entire fusion framework is trained on the TNO dataset in two stages: a pre-training phase and a fine-tuning phase. During pre-training, we select 46 image pairs, which are converted into grayscale. To fully leverage the gradient and pixel information from each image, 1,410 image blocks of size 64 × 64 are cropped from the source images. The Adam optimizer is used with a learning rate of 0.0001 and a batch size of 30. During this phase, only the fundamental loss is employed to update the network parameters. In the fine-tuning phase, the dataset consists of 18 images from the TNO dataset containing significant masks. As in the pre-training phase, 1,410 image blocks of size 64 × 64 are extracted. A contrast-constrained loss is applied, utilizing a positive sample alongside three negative samples. The network is trained for 5 epochs, using the same optimizer, learning rate, and batch size as in the pre-training phase. In this stage, both fundamental and contrast losses are utilized to update the network weights.

#### Comparison models and evaluation metrics.

To assess the effectiveness of the proposed algorithm, we compare it with six state-of-the-art fusion algorithms, as well as foundational models, for a total of seven comparison models: SwinFusion [30], U2Fusion [41], RFN [42], DenseFuse [15], LRRNet [43], DIDFuse [44], and CoCoNet [36]. The experimental parameters of the comparison methods are fine-tuned based on the settings provided in the original papers and adapted to the laboratory configuration.

For quantitative evaluation, we employ six metrics to measure fusion performance: Average Gradient (AG), Entropy (EN), Standard Deviation (SD), Spatial Frequency (SF), Visual Information Fidelity (VIF), and Structural Content Discrepancy (SCD).

#### Results and analysis on TNO dataset.

**Fig 6:**
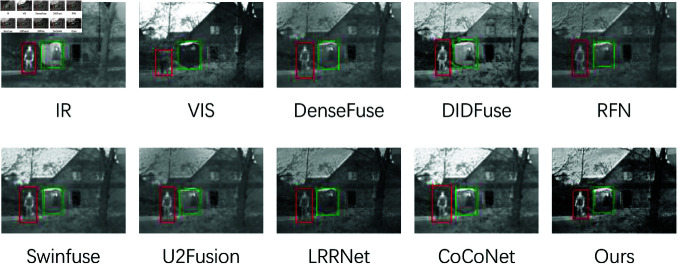
The qualitative comparisons on TNO 01.png.

**Fig 7:**
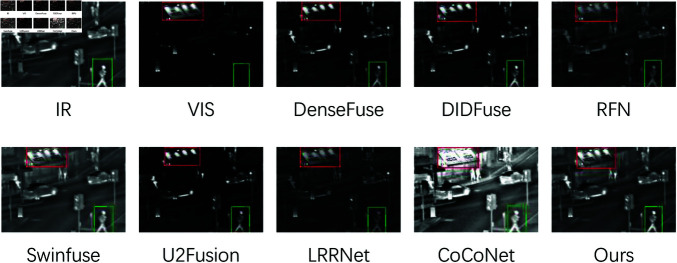
The qualitative comparisons on TNO 02.png.

Qualitative Comparisons on TNO: The introduction of LCFM and LMGC significantly enhances our results, providing well-defined features such as figures, bushes, and road signs (highlighted by red and green boxes) with clear background texture. As seen in the first image (Fig 6), the salient object (the figure in the red box) is more distinct due to the edge enhancement facilitated by the multilevel feature integration module. While DenseFuse and DIDFuse also deliver clear thermal features, their images lack brightness, which diminishes their overall visual quality. U2Fusion and RFN fail to render a clear target, resulting in blurry depictions of humans. In contrast, our fusion images preserve vivid texture details. In the second (Fig 7), the fusion results from CoCoNet and SwinFusion exhibit low contrast, with the target (the human in the green box) appearing blurred and indistinct. Other algorithms show limited texture detail and poor visualization under low light conditions. Our model achieves the optimal balance between saliency and vivid detail preservation.

Quantitative Comparisons on TNO: As shown in Table 1, the red highlights indicate the highest value in each metric. This demonstrates that our proposed network outperforms the other seven algorithms. Specifically, on the TNO dataset, our algorithm achieves the best results for EN, SCD, and AG, indicating superior fusion of texture details and appropriate contrast. While our method ranks second for VIF and SF, the performance is only marginally lower than the top-ranked algorithm. It ranks third for SD, but the difference compared to the top two algorithms is minimal, suggesting potential for further improvement.

**Table 1:** Performance comparison of various methods on the TNO and Roadscene datasets. EN: Entropy, SD: Standard Deviation, VIF: Visual Information Fidelity, SF: Spatial Frequency, AG: Average Gradient, SCD: Structure Consistency Deviation, SSIM: Structural Similarity, Qabf: Fusion Quality Index.

	TNO								Roadscene							
Method	EN	SD	VIF	SF	AG	SCD	SSIM	Qabf	EN	SD	VIF	SF	AG	SCD	SSIM	Qabf
DenseFuse	6.4572	8.3479	0.6722	0.0326	2.5764	1.5794	0.268	0.281	7.0279	9.8652	0.7628	0.0368	3.0981	1.6528	0.298	0.263
DIDFuse	6.9832	10.0915	**0.8172**	0.0468	5.2389	1.8034	0.266	0.288	7.2380	10.3480	**0.8504**	0.0428	4.0921	1.6923	0.301	0.278
RFN	7.0429	9.9320	0.7743	0.0239	2.7643	1.5820	0.301	0.279	7.3420	9.5490	0.8044	0.0279	2.4590	1.7732	0.319	0.282
SwinFusion	6.8753	9.5628	0.7763	**0.0562**	4.2981	1.5290	0.299	0.281	6.9342	10.2390	0.8135	0.0423	3.0918	1.6583	0.289	0.273
U2Fusion	6.4380	9.3489	0.6270	0.0347	2.5881	1.3780	0.298	0.275	7.0234	10.1246	0.7392	0.0423	4.2370	1.4593	0.297	0.261
LRRNet	6.8583	9.3091	0.7123	0.0478	3.5692	1.5890	0.312	0.327	6.9023	9.3780	0.8318	0.0428	3.9820	1.5638	0.362	0.362
CoCoNet	7.7552	**10.153**	0.6532	**0.0639**	5.6893	1.7378	0.472	0.427	7.6322	10.3567	**0.8543**	**0.0717**	5.3480	1.8023	0.499	0.427
ours	**7.8928**	9.9662	0.7962	0.0593	**6.0327**	**1.8229**	**0.489**	**0.482**	**7.8239**	**10.4162**	0.8532	0.0653	**5.6371**	**1.8439**	**0.523**	**0.452**

#### Results and analysis on Roadscene dataset.

**Fig 8:**
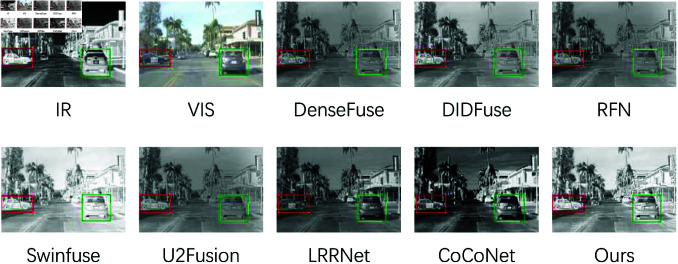
The qualitative comparisons on Roadscene 04424.png.

Qualitative Comparisons on Roadscene Dataset: In Roadscene.04424 (Fig 8), the fusion results from RFN, DenseFuse, and U2Fusion (vehicles in the red and green boxes) exhibit unclear edges and low contrast. DIDFuse produces poorly lit significant targets with blurred textures, while the SwinFusion result is overly bright, leading to a less visually appealing image. The fusion images from LRRNet and CoCoNet appear darker but retain clear textures. In contrast, our method generates brighter and more distinct significant targets (pedestrians in the red boxes) with clear background textures, demonstrating the effectiveness of our cross-fertilization module and multi-branch feature interaction module in capturing edge detail information.

Quantitative Comparisons on Roadscene Dataset: Table 1 presents a quantitative comparison of the RoadScene dataset. Our method achieves the best results for SD, EN, AG, and SCD, confirming the superior fusion results of our approach. For the remaining metrics, our algorithm ranks second, with only a 5% reduction compared to the top-performing method. As shown in Fig 7, we quantitatively compare our algorithm to seven existing fusion methods using six metrics across 10 image pairs from the RoadScene dataset. Our method ranks highest in all metrics except VIF and SF, affirming that our fusion strategy successfully retains the most information and achieves the best overall fusion performance.

**Fig 9:**
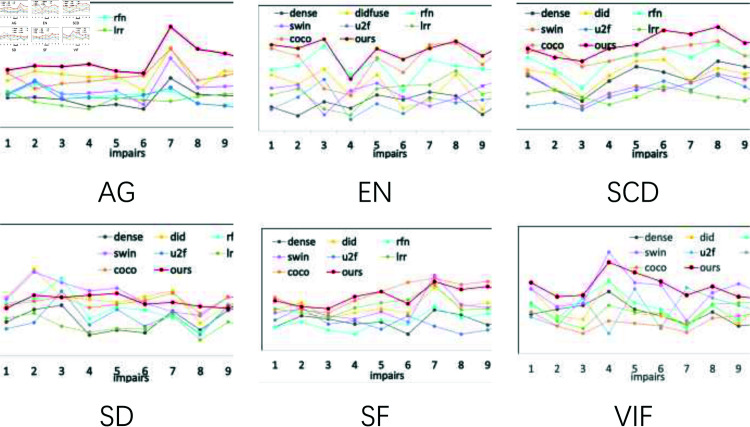
Using six metrics to quantitatively compare DMCM with six methods on the RoadScence dataset.

As shown in Fig 9, we use six metrics to compare the algorithm in this paper with the seven existing fusion methods (10 image pairs) on the RoadScence dataset for quantitative comparison. The figure shows that except for VIF and SF all other metrics perform best, indicating that this paper’s algorithm fuses the results with the most information and the best fusion strategy.

To further evaluate the real-time performance and resource consumption of the algorithm across different hardware environments, comparative experiments were conducted on a variety of platforms. High-Performance GPU Environment: The representative device was the NVIDIA RTX 3090, known for its exceptional computational power and abundant memory, making it ideal for server-side batch processing and high-performance tasks.Use Case: Large-scale image fusion tasks, such as real-time surveillance systems or cloud-based image processing services. Mid-Range GPU Environment: The representative device was the NVIDIA GTX 1650, which offers low power consumption and adequate computational capability, making it suitable for lightweight tasks.Use Case: Small-scale image fusion tasks on personal computers or portable workstations. Embedded Computing Environment (High-End Embedded Device): The representative device was the NVIDIA Jetson Xavier NX, specifically designed for embedded AI applications. It delivers relatively high computational performance, supports CUDA acceleration, and has low power consumption.Use Case: Real-time processing for drones, smart cameras, and in-vehicle systems.Embedded Computing Environment (Low-End Embedded Device): The representative device was the Raspberry Pi 4 (with 4GB of memory), characterized by its limited computational resources and low power consumption, making it ideal for resource-constrained edge devices. Use Case: Oﬄine processing tasks with low real-time requirements, such as sensor fusion and data collection.

Experimental results demonstrated that the NVIDIA RTX 3090 excelled in real-time performance, with the shortest processing time and lowest latency. However, it consumed the most memory and power. In contrast, the Raspberry Pi 4 had the lowest memory and power usage, albeit with significantly reduced real-time performance. Therefore, the choice of hardware environment for deploying the algorithm should be tailored to the specific requirements of the application.

**Table 2:** Hardware performance comparison across different platforms.

Device	Time (S)	Memory (GB)	Power Consumption (W)	Frame Rate (FPS)	Latency (ms)
NVIDIA RTX 3090	**0.073**	10	350	**60**	8
NVIDIA GTX 1650	0.084	6	75	40	25
NVIDIA Jetson Xavier NX	0.093	8	10	20	60
Raspberry Pi 4	1.023	**4**	**4**	10	120

To analyze the adaptability and robustness of the multi-dimensional fusion branches across different scenarios, comparative experiments were conducted under the following conditions:Daytime Scenario: Characterized by uniform lighting and rich details, but complex backgrounds may interfere with target features.Adaptability Analysis: The multi-branch design enhances texture details and suppresses interference from the complex background, ensuring that target features are prominently represented.Nighttime Scenario: The lighting is insufficient, and the contrast between target areas and the background is low, with noise potentially being more prominent.Adaptability Analysis: The module effectively suppresses noise and extracts hidden high-frequency information, improving the visibility of nighttime targets (e.g., vehicles, pedestrians).Low-Light Scenario: This scenario features significant lighting variations (e.g., alternating light and dark regions, indoor-to-outdoor transitions) with stark contrasts in brightness between different areas.Adaptability Analysis: By incorporating cross-attention and spatial attention mechanisms, the module balances feature representation between bright and dark regions, reducing the impact of uneven lighting on fusion quality.

We conducted experiments on the TNO dataset across these various scenarios to assess the adaptability and robustness of the proposed method, as shown in Table 3. The results demonstrate that, in daytime scenarios with abundant lighting, the four metrics were the highest. In contrast, the metrics slightly decreased in nighttime and low-light scenarios, indicating that the proposed algorithm exhibits strong robustness and adaptability across different environments.

**Table 3:** Performance metrics for different lighting scenarios.

Scenario	EN	SD	VIF	SF
Daytime Scenario	**7.8942**	**9.9676**	**0.7974**	**0.0598**
Nighttime Scenario	7.8738	9.9537	0.7863	0.0573
Low Light Scenario	7.8863	9.9626	0.7928	0.0583

### Ablation studies

To assess the performance of each module, ablation experiments were conducted on the TNO and Roadscene datasets. In the first set of experiments, the Swiftformer module was removed while keeping the other modules intact, referred to as ST. In the second set, the multidimensional hybrid spatial attention was excluded, referred to as MHSA. The third set involved replacing the lightweight cross-attention fusion module with the channel attention module, referred to as LCFM. The fourth set replaced the dual-branch multilevel fusion module with the channel attention module, referred to as DMFM. The fifth set replaced the lightweight multi-scale grouped convolution with standard convolution, referred to as LMGC. The sixth set represents the proposed model, denoted as Ours.

Subjective Evaluation: As shown in the Table 4, removing the Swiftformer module results in a significant decrease in performance metrics on both the TNO and Roadscene datasets, highlighting the importance of global feature extraction for effective image fusion. The fusion metrics in the second to fourth experiments also show slight declines, confirming that the modules introduced in this study enhance image fusion performance. The removal of the lightweight multi-scale grouped convolution causes only a minor reduction in the metrics, suggesting that its capacity for local feature extraction is comparable to that of standard convolution.

**Table 4:** Performance comparison of different models on TNO and RoadScene datasets.

	TNO						RoadScene					
**Model**	EN	SD	VIF	SCD	SSIM	Qabf	EN	SD	VIF	SCD	SSIM	Qabf
I	6.4721	7.3482	0.6573	1.6483	0.352	0.392	6.6728	9.2416	0.7351	1.7263	0.433	0.337
II	**7.2368**	7.4522	0.6758	1.6783	0.472	0.416	7.7478	9.4025	0.8126	1.7786	0.473	0.402
III	6.3621	7.6721	0.7134	1.6834	0.342	0.367	6.8356	9.3776	0.7035	1.7362	0.424	0.334
IV	6.5828	7.7364	0.6728	1.6573	0.387	0.374	6.9743	9.4102	0.7429	1.7429	0.452	0.342
V	7.1242	**8.3472**	0.7284	1.7365	0.452	0.437	7.4051	9.8784	0.8105	1.8126	0.493	0.427
VI	**7.8827**	**9.9582**	**0.7936**	**1.8217**	**0.489**	**0.482**	**7.8214**	**10.4136**	**0.8521**	**1.8437**	**0.523**	**0.452**

**Fig 10:**
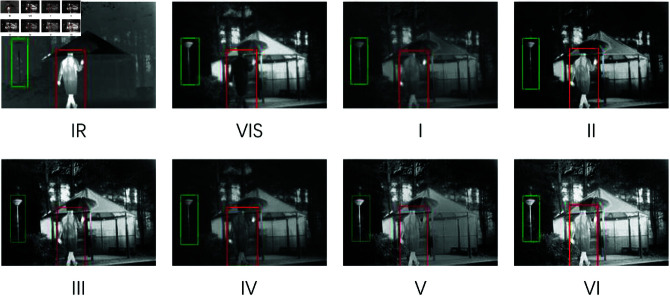
Visual comparison of different model architectures.

Objective Evaluation: In Fig 10, the absence of Swiftformer results in a network that lacks global structural features and exhibits reduced image brightness. The second set, which lacks NHSA, produces images with diminished clarity. The third set, without LCFM, results in darker image regions, particularly affecting character visibility. The fourth and fifth sets, which lack edge enhancement branches and multi-scale information fusion, lead to blurred texture edges, demonstrating the superior capability of the proposed algorithm in capturing detailed features. The sixth set, representing the proposed model, achieves clear character and streetlight contours, well-defined background textures, and balanced illumination.

**Fig 11:**
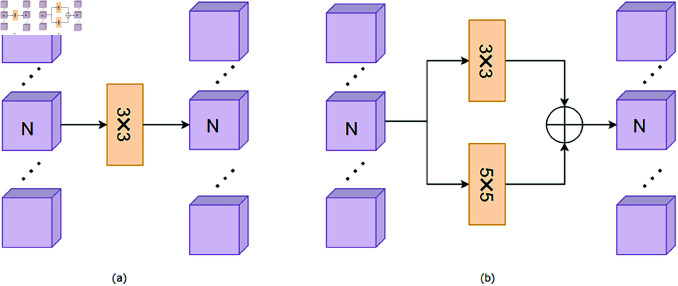
Ablation experiments of stacked small convolutional nuclei legend: (a) The first experiment used a single 3×3 convolution (b) The second experiment utilized parallel 3×3 and 5×5 convolutions.

To demonstrate the effectiveness of stacking small convolution kernels, we conducted ablation experiments on the TNO dataset. The first experiment used a single 3×3 convolution, keeping other parts unchanged (see Fig 11(a)). The second experiment utilized parallel 3×3 and 5×5 convolutions (see Fig 11(b)). The third experiment employed our proposed stacked small convolution kernel method.As shown in Table 5, our method achieved the best performance across all four metrics, demonstrating the superiority of stacked small convolution kernels for image fusion.

**Table 5:** Performance metrics comparison across different methods.

Method	EN	SD	VIF	SF
single 3×3 convolution	7.7128	9.8736	0.7638	0.0573
parallel 3×3 and 5×5 convolutions	7.7783	9.9162	0.7726	0.0583
ours	**7.8921**	**9.9648**	**0.7962**	**0.0594**

**Fig 12:**
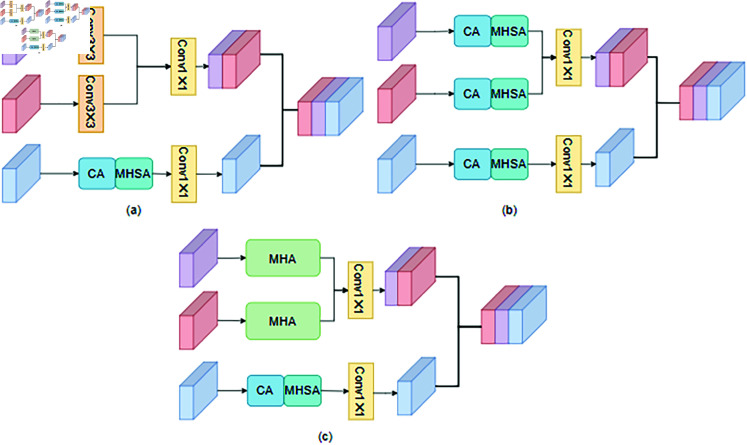
Ablation experiments with cross-attention legend: (a) standard 3×3 convolutions for both branches (b) channel attention for both branches (c) multi-head attention for both branches.

To demonstrate the superiority of the proposed cross-attention mechanism, we conducted comparative and ablation experiments on the TNO dataset.In the comparative experiments, we compared cross-attention with other attention mechanisms and convolution operations. Specifically:In the first experiment, cross-attention was replaced with standard 3×3 convolutions for both branches (see Fig 12(a)). In the second experiment, cross-attention was replaced with channel attention for both branches (see Fig 12(b)). In the third experiment, cross-attention was replaced with multi-head attention for both branches (see Fig 12(c)). For the ablation experiments:In the first experiment, differential attention was removed, with all other components unchanged.In the second experiment, common attention was removed, with all other components unchanged.The third experiment used the proposed method with both attention mechanisms intact.

As shown in Table 6, the proposed cross-attention mechanism achieved the best performance across all four metrics in the comparative experiments, demonstrating its advantages over other attention mechanisms and convolution operations.In the Table 7, removing either differential or common attention resulted in a decline in performance metrics, highlighting that both types of attention are indispensable.

**Table 6:** Performance metrics comparison across different methods.

Method	EN	SD	VIF	SF
3×3 convolutions	6.8972	8.4728	0.7182	0.0428
channel attention	7.5429	9.8728	0.7289	0.0572
multi-head attention	6.7628	8.2781	0.6829	0.0437
ours	**7.8921**	**9.9648**	**0.7962**	**0.0594**

**Table 7:** Performance metrics comparison across different methods.

Method	EN	SD	VIF	SF
w/o differential attention	7.8263	9.9182	0.7463	0.0537
w/o common attention	7.8028	9.8521	0.7372	0.0517
ours	**7.8921**	**9.9648**	**0.7962**	**0.0594**

**Fig 13:**
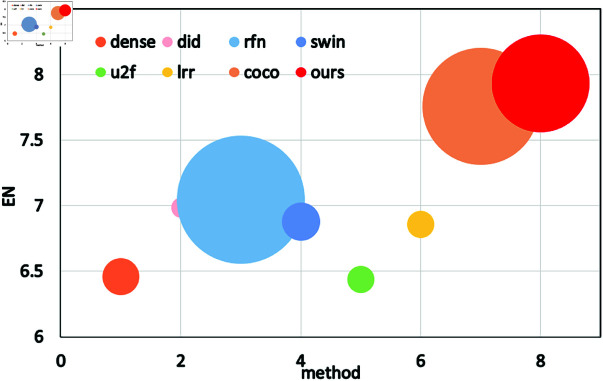
The number of model parameters and the EN values on TNO dataset.

### The number of training parameters

In addition to qualitative and quantitative analyses, model size and runtime speed are critical factors in practical applications. Consequently, we evaluate the memory consumption and computational efficiency of the proposed model. It is important to note that the results presented in this section may differ slightly from those in the original paper due to variations in platform settings, hyperparameters, and other factors. Specifically, FLOPs and the number of training parameters are computed using an input size of 64×64 pixels. For runtime measurements, we randomly selected ten images from the TNO dataset to calculate the average execution time.

Fig 13 and Table 8 presents the quantitative results for model size, FLOPs, and runtime for several state-of-the-art methods. Our model demonstrates superior speed, outperforming all methods except DIDFuse and CoCoNet. The incorporation of spatial attention and the SwiftFormer module increases the number of parameters compared to DIDFuse and LRRNet. However, by employing lightweight multi-scale grouped convolution and the lightweight cross-fusion module, our algorithm reduces the parameter count by 40% relative to CoCoNet. Although our runtime is 15% longer than CoCoNet, our model still ranks third in overall performance. Furthermore, while the enhanced multi-branch feature interaction module results in a larger number of parameters, it effectively extracts more edge and texture information, leading to clearer fusion results.

**Table 8:** Performance comparison of running times.

Method	DenseFuse	DIDFuse	RFN	U2Fusion	SwinFuse	LRRNet	CoCoNet	Ours
TP(M)	0.925	**0.261**	10.936	0.659	0.974	0.492	9.130	6.353
FLOPS(G)	497.06	**18.71**	676.09	366.34	471.04	134.79	115.37	178.39
Times(s)	0.124	0.055	0.239	0.123	1.345	0.079	**0.052**	0.073

### Infrared-visible object detection

Target detection is a well-established and extensively studied task in advanced computer vision. With the continual improvement of multimodal datasets, their capacity to capture and reflect semantic information has become increasingly important for evaluating multimodal image fusion techniques. This subsection examines the impact of image fusion on target detection. Implementation Details: Experiments were conducted on the $M^{3}FD$ dataset [45] using the state-of-the-art YOLOv5 [46] detector. The detector’s configuration was kept consistent with its original settings, and all quantitative results were directly derived from the test code. The quantitative comparison results, obtained by comparing six detectors, are presented in Table 9. The metric mAP@.5 refers to the mean Average Precision (mAP) at an Intersection over Union (IoU) threshold of 0.5, while the other values represent the Average Precision (AP) for the respective classes. In terms of mAP@.5, which is the primary metric of interest, the proposed method demonstrates superior performance.

**Table 9:** Performance comparison of detection on different object classes.

Model	Bus	Car	Lamp	Motor	People	Truck	mAP@0.5
IR	78.23	87.39	70.12	61.24	78.33	65.27	74.13
VIS	78.29	90.39	87.39	68.35	70.14	70.28	77.26
DenseFuse	94.32	88.17	90.27	68.83	65.83	71.93	80.47
DIDFuse	79.63	**92.49**	84.37	68.39	**79.36**	68.63	78.93
RFN	78.17	91.83	84.38	**72.91**	78.39	68.73	79.28
SwinFusion	94.39	87.34	89.74	67.83	65.32	74.02	80.24
U2Fusion	**95.15**	88.84	88.94	68.52	65.38	72.63	80.12
LRRNet	77.81	86.39	83.68	67.73	62.38	69.63	73.29
CoCoNet	94.23	88.50	**90.63**	71.63	65.01	73.81	80.63
Ours	94.69	88.72	90.48	71.93	71.63	**74.21**	**80.79**

## Conclusion

This paper introduces a multi-scale image fusion algorithm with a two-branch, multi-level feature fusion structure to achieve effective fusion between infrared and visible images. A lightweight multi-scale grouped convolution is proposed to reduce network parameters while extracting both local features and multi-scale information. Additionally, the multi-level feature integration module is improved by incorporating an edge feature enhancement branch and replacing the channel attention mechanism for shallow feature extraction with a lightweight cross-attention fusion module. This adjustment, tailored for multimodal fusion, enhances edge information extraction and enables more effective fusion of shallow features from both modalities. Furthermore, an improved spatial attention mechanism is introduced to incorporate a multi-dimensional fusion branch, allowing for more comprehensive spatial information fusion. Extensive experiments conducted on publicly available datasets demonstrate that the proposed algorithm consistently outperforms existing algorithms in both objective and subjective evaluations. While running efficiency experiments suggest that further optimization is needed in terms of processing speed, the algorithm still achieves competitive performance in target detection tasks when compared to other methods.
